# Perturbations in levels of essential metals less severe in Parkinson’s disease without dementia than in Parkinson’s disease dementia

**DOI:** 10.1093/mtomcs/mfaf006

**Published:** 2025-02-11

**Authors:** Melissa Scholefield, Stephanie J Church, Garth J S Cooper

**Affiliations:** Division of Cardiovascular Sciences, School of Medical Sciences, Faculty of Biology, Medicine and Health, Manchester Academic Health Science Centre, The University of Manchester, Manchester M19 9NT, United Kingdom; Division of Cardiovascular Sciences, School of Medical Sciences, Faculty of Biology, Medicine and Health, Manchester Academic Health Science Centre, The University of Manchester, Manchester M19 9NT, United Kingdom; Division of Cardiovascular Sciences, School of Medical Sciences, Faculty of Biology, Medicine and Health, Manchester Academic Health Science Centre, The University of Manchester, Manchester M19 9NT, United Kingdom; School of Biological Sciences, Faculty of Science, University of Auckland, Auckland 1142, New Zealand

## Abstract

It is currently unknown why some individuals with Parkinson’s disease (PD) go on to develop dementia [Parkinson’s disease dementia (PDD)], whereas others do not. One possibility is differences in susceptibility to metallomic dysregulation. A previous study of the PDD brain identified substantive perturbations in metal levels, including severe multiregional decreases in Cu. The current work uses the same methods to ascertain whether this metallomic dysfunction is also present in the PD brain. To do this, tissue from 9 PD cases free of cognitive decline and 15 equivalent controls was obtained from 7 brain regions. Levels of Na, Mg, K, Ca, Mn, Fe, Cu, Zn, and Se were quantified using inductively coupled plasma mass spectrometry (ICP-MS). Multiple linear regression analysis was used to determine any potential confounder effects. Results were compared with those previously obtained for PDD. It was found that decreased Cu in the medulla was the only statistically significant case-control difference observed in the PD brain; this contrasts markedly with the widespread metallic dysfunction observed in PDD. PD and PDD cases were well separated by PCA analysis. In the PD cohort, tau Braak stage correlated with Cu concentrations in several regions, but these correlations were not retained when including PDD cases. There is a marked difference in the metallomic profiles of PD and PDD, with an almost complete lack of metallic involvement observed in the former. This resistance to metallomic dysfunction may contribute to resilience against cognitive impairment in individuals with PD who do not develop dementia.

## Graphical Abstract

**Graphical Abstract:**
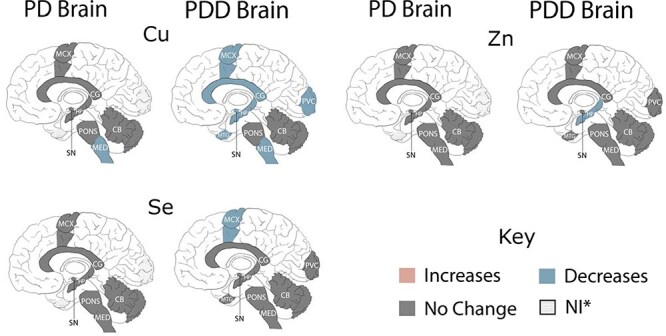
The PD brain shows much lower overall metallomic involvement than the PDD brain, with decreased Cu in the medulla oblongata being the only metallic alteration observed across seven investigated brain regions.

## Background

Parkinson’s disease (PD) is the second most common age-related neurodegenerative disease, with an estimated global incidence of 3.8 cases per 1000 persons in 2023 [1]. Clinically, PD is primarily characterized by parkinsonian motor symptoms, including bradykinesia, rigidity, and resting tremor. However, non-motor symptoms such as rapid eye movement sleep behaviour disorder (RBD), constipation, orthostatic hypotension, loss of smell, and apathy, among others, may occur years before the onset of motor dysfunction [2].

A definitive diagnosis of PD is made *postmortem* based on neuropathological examination, primarily by the observation of dopaminergic neuronal loss in the brain, most heavily affecting the substantia nigra pars compacta (SNpc), but also affecting many other regions of the brain such as the medulla oblongata, pons, hippocampus (HP), cingulate gyrus (CG), and motor cortex (MCX), etc., depending on the severity of the disease upon death. Dopaminergic cell loss is usually accompanied by the deposition of misfolded α-synuclein-containing protein accumulations known as Lewy bodies, and Alzheimer’s disease (AD)-type neuropathologies such as tau tangles and amyloid-β inclusions can also be common, particularly in individuals with cognitive decline [3]. Unfortunately, despite great efforts, therapeutics aiming at treating PD through the removal of Lewy bodies have not yet shown significant beneficial effects on the progression of the disease, and there are still no available treatments capable of slowing, preventing, or reversing the course of the condition [4]. At present, the gold-standard treatment of PD remains the administration of l-dopamine (l-DOPA), a dopamine precursor that temporarily restores the dopamine lost as a result of dopaminergic neuronal death; however, even this treatment becomes less and less effective over time, with many side effects including hallucinations, psychosis, and the development of l-DOPA-induced dyskinesia [5]. As such, the identification of alterative pathogenic mechanisms in and potential therapeutic targets for PD remain leading goals in PD research.

One of these alternative disease mechanisms may be metallic dysfunction in the PD brain. There have been a number of studies investigating this area of research, primarily focusing on copper and iron, but with isolated studies also covering other metals such as zinc, manganese, and selenium. The most-often researched of these metals in PD is iron, which has received substantial attention, although results have varied between studies; e.g. multiple groups have observed increased iron in several regions of the PD brain, including the substantia nigra (SN; [6–14]), the globus pallidus (GP; [8]), and putamen (PUT; [12]), while others have reported no changes in the cortex [6], GP [7, 12, 14, 15], PUT [7, 14–16], occipital cortex (OCC; [9]), frontal gyrus (FG; [6]), caudate nucleus (CN; [12, 14–16]), HP [16], thalamus [15, 16], amygdala [16], and even the SN itself [15, 16]. Copper has been observed to be decreased in the PD SN [6, 9, 17, 18], CN [17], and locus coeruleus (LC; [17]), while investigations of the cortex [6] and OCC and FG [9] have found no changes. A single study by Genoud et al. observed no differences in Zn, Mn, or Mg in the SN, OCC, or FG of the PD brain [9], but these metals have generally received very little coverage in studies of the PD brain. Metallomic dysfunction has also been observed in peripheral matrices such as the plasma or serum in individuals with PD, but observations vary greatly between studies and very often do not reflect measurements within the brain [19].

Many individuals with PD will live with the condition for many years, and for every year living with the disease, the risk of also developing comorbid dementia (Parkinson’s disease dementia; PDD) increases, with those surviving for 20 years having an 80% chance of showing symptoms of cognitive decline [20, 21]. However, not every individual with PD goes on to develop concurrent dementia, and it is unknown what puts an individual at risk of cognitive decline and why others are more resilient—particularly after multiple decades of living with PD. Many studies of metals in PD have either not focused on PDD or have not taken cognitive status into account when selecting subjects for study cohorts. In light of this prior limitation, our group performed a multiregional, multielement study of nine essential metals across nine regions of the PDD brain in order to determine the presence of metallic perturbations in PDD [22]. We found that there were widespread decreases in copper levels in PDD, affecting almost every investigated region except the LC and cerebellum (CB). Fairly widespread decreases in Mn as well as more localized decreases in zinc, selenium, magnesium, and potassium were also observed. By contrast, we observed no changes in Fe levels between PDD cases and controls.

The aim of the present study was to perform a comparable study in the PD brain, with tissues donated by subjects confirmed to have shown no presence of cognitive decline during their lifetime. By doing so, we may be able to discover some indication of whether metallic dysfunction differs between the PD and PDD brain.

## Materials and methods

### Reagents

Except where otherwise stated, all reagents were obtained from Sigma-Aldrich (UK).

### Acquisition of human brain tissues from PD cases and controls

Brain tissues were obtained from the Multiple Sclerosis and Parkinson’s Tissue Bank, Imperial College London, UK, through the Medical Research Council (MRC) brain bank network from nine PD cases and nine age, sex, and postmortem delay (PMD)-matched controls. Tissues were obtained for seven brain regions, including the SN, pons at the level of the LC, CG (Brodmann area 24), HP, medulla (MED), MCX (Brodmann area 4), and CB. These regions were selected to (a) cover regions of the brain associated with high, moderate, and relatively low levels of neurodegeneration at end-stage PD and (b) to be directly comparable to a previous analysis of the PDD brain; previously analyzed PDD tissues were also obtained from the Multiple Sclerosis and Parkinson’s Tissue Bank for these seven regions.

Data obtained for cases and controls included age, sex, brain weight, PMD, tau Braak stage, α-synuclein Braak stage, cause of death, comorbidities, presence and severity of small vessel disease (SVD), and medications taken, where available; this data is all included in Supplementary Material A1. For some cases, the duration of disease was also obtained. No case or control was recorded as having amyloid angiopathy. Cases and controls were sex, age, PMD, and brain weight-matched, and were also matched across these variables with a previous PDD cohort to ensure comparability.

### Ethics approval and consent to participate

All donors (and/or their families, when applicable) gave informed consent for the donation of the brain tissues used in this study. The collection of tissues was approved by the MRC brain bank network (Ref. No. 18/WA/0238); the current study was reviewed, approved, and carried out in compliance with the approval made by Manchester REC (09/H0906/52+5).

### Diagnosis and severity of human cases

PD and PDD cases were diagnosed by neuropathologists at the Multiple Sclerosis and Parkinson’s Tissue Bank according to neuropathological analysis and clinical diagnoses made during the individual’s lifetime, based on medical records. A neuropathological diagnosis of either PD or PDD required an α-synuclein Braak stage of three or more [23]; both α-synuclein and tau Braak staging for all cases and controls can be found in Tables S1 and S2. For the purposes of this study, we aimed to keep tau Braak stages as low as possible, at two or below, in order to minimize the effects of comorbid AD pathology. Information on the duration of the disease was obtained where available.

Cases with PD were confirmed to be free of cognitive decline, including mild cognitive impairment (MCI), while cases with PDD were confirmed to show cognitive decline during their lifetime.

### Tissue dissection

Flash-frozen brain tissue was stored at −80°C until analysis. On the experimental day, tissues were thawed slightly on ice to allow dissection. Tissues were sectioned into 50 mg (±5%) wet weight for inductively coupled plasma mass spectrometry (ICP-MS) using a metal-free ceramic scalpel to prevent contamination from trace metals and placed into ‘Safe-Lok’ microfuge tubes (Eppendorf AG, Hamburg, Germany).

### ICP-MS

Freshly dissected samples were briefly centrifuged before being dried for 6 hours to a constant weight (∼10 mg; individual sample wet and dry weights are provided in Supplementary Material B) using a Savant Speedvac^™^ (Thermo Fisher Scientific, Waltham, MA, USA) at room temperature (RT). A digestion mix was created by mixing concentrated nitric acid and an internal standard mix containing a known concentration of each metal to be measured at a ratio of 20:1. Following drying, 200 µl of this digestion mix was added to each sample, and digestion was performed by heating in a block at 60°C for 30 min followed by 100°C for 3.5 hours. Following this, 100 µl of each sample was added to 5 ml of MS-grade water (1:50). Digestion blanks containing only nitric acid were digested following the same method.

Following digestion, samples and blanks were refrigerated overnight at 4°C before undergoing ICP-MS analysis with a 7700× ICP-MS spectrometer (Agilent, Santa Clara, CA, USA) equipped with a MicroMist nebulizer and Scott double-pass spray chamber (Glass Expansion, Melbourne, Australia) and nickel sample and skimmer cones. Samples were separated into batches of either one or two regions, with multielement calibration using calibration standard dilutions (see Supplementary Material B for all raw data and values for blanks and standard curves). All elements were standardized against scandium, with the exception of zinc and selenium, which were standardized against germanium. All regions were run in triplicate.

The manufacturer’s recommendations were followed for the selection of operation mode, integration times, and internal standard assignments. Samples were introduced to the instrument using an integrated autosampler (Agilent, Santa Clara, CA, USA). The concentrations of eight essential metals (Na, Mg, K, Ca, Mn, Fe, Cu, and Zn) and the metalloid Se were determined. All elements were analyzed using helium as the collision gas; selenium was analyzed in high-energy helium mode (10 ml/min helium) due to its potential state as a polyatomic ion, and all other elements were analyzed using standard helium mode (5.0 ml/min helium). Results were excluded from analysis where the highest blank value for any given analyte during a run was ≥15% of that of the lowest sample value. Sample concentrations were normalized by dry weight and quantified using the standard curve created using the calibration standard dilutions.

### ICP-MS data analysis

Case-control age, sex, brain weight, and PMD were compared using nonparametric Mann–Whitney *U* tests. Mean metal values [±standard deviation (SD) and with 95% confidence intervals (CIs)] were calculated, normalized to the sample’s dry weight, and differences between cases and controls determined by Mann–Whitney *U* tests due to the small sample size precluding the appropriate use of data distribution tests. Shannon diversity indices (*S*-values, also termed surprisal scores) were also calculated by taking the negative base 2 log of the *P*-value. CIs were calculated using the following equation:


(1) \begin{eqnarray*}
CI = SE*Z\left( {0.95} \right),
\end{eqnarray*}

where SE = the standard error and *Z* (0.95) = the *z*-score corresponding to a confidence level of 0.95. Mann–Whitney *U* calculations were performed using GraphPad v8.1.2 (Prism, La Jolla, CA, USA). *P*-values <.05 were considered significant. Comparisons of metal levels across different regions in only control or only PD brains were also carried out using Kruskal–Wallis tests in GraphPad v.8.1.2.

### Sensitivity analyses

In order to assess whether the interpretation of the data obtained in the current study was appropriate and robust, a sensitivity analysis was performed for every significant (*P* < .05) case-control difference in metal levels. For both individual runs and the mean values of all three replicate runs taken together, the risk ratio (RR), *E*-value, and effect size were determined. An explanation of these is given below. A detailed explanation of these measures is given in [24]; a more concise explanation is given below.

The RR is determined by the following equation:


(2) \begin{eqnarray*}
RR = \left( {\frac{a}{b}} \right)/\left( {\frac{c}{d}} \right),
\end{eqnarray*}

where ***a*** = the number of case values >95% upper CI limit of the controls (or <95% lower CI limit where significant *decreases* were observed in cases), ***b*** = number of cases, ***c*** = number of control values >95% upper CI limit of the controls (or <95% lower CI limit where significant *decreases* were observed in cases), and ***d*** = number of controls. RRs of >3 were considered to be robust. In the case of null values in the calculation of RRs, the null values were assigned a value of 0.5.

*E*-values were calculated for RRs as well as for the upper and low confidence limits of the RRs. The *E*-value was calculated using Equation (3):


(3) \begin{eqnarray*}
E\,\,\textit{value} = RR + \textit{sqrt}\,\,\left( {RR\,\,x\,\,\left( {RR - 1} \right)} \right).
\end{eqnarray*}

In the calculation of *E*-values for RR < 1, the inverse of the RR was first taken. *E*-values were also calculated for the CIs of the RRs; if the range of the CIs crossed 1.0, then the *E*-value was determined to be 1.0; otherwise, *E*-values were calculated according to Equation (3), substituting RR for the CI of the RR, substituting RR for the CI closest to 1.0.

The effect size was here determined using Glass’ Delta:


(4) \begin{eqnarray*}
\textit{Glass}^{\prime}\,\,\Delta \,\, = ({M_1} - {M_2})/\sigma \textit{control},
\end{eqnarray*}

where *M*_1_ = mean case value, *M*_2_ = mean control value, and σcontrol = SD of the control group; *M*_1_ and *M*_2_ were reversed in case of significant decreases. Glass’ delta was used as the group sample sizes were equal, but their SDs were unequal. Effect size values of 0.2–0.5 were considered small, values between 0.50 and 0.80 were considered of medium size, values between 0.80 and 1.30 were considered large, and effect sizes >1.30 were considered very large.

### PCA and PLS-DA

To determine whether the findings of the metallomic analysis were able to separate PD and PDD cases, principal component analysis (PCA) and principal least squares-discriminant analysis (PLS-DA) were carried out for each investigated brain region. PCA and PLS-DA analyses were carried out in MetaboAnalyst with log transformation and auto-scaling (mean-centring and division of the SD of each variable). Metals with variable importance in projection (VIP) scores of >1.5 in the PLS-DA model were considered to contribute to case–control separation in that region.

### Analysis of confounding variables

Any potential confounding effects of age, tau Braak stage, and PMD on metal concentrations in PD cases and controls were determined by carrying out multiple linear regressions with 10% FDR correction firstly for the averaged concentrations of each metal for each sample across all regions and secondarily for individual regions. Results with *P* < .05 are represented as individual regressions for clarity. Differences between metal concentrations in males and females were investigated using two-way ANOVA, taking PD status into account.

### Comparison with PDD

The results of the PD metallomic analysis were compared with those obtained for PDD in a previous study [22, 25]. This was first done simply by comparing significant case-control findings from each disease study. Following this, PCA and PLS-DA were carried out to determine if metallomic data from regions investigated in both PD and PDD could be used to separate these two conditions. Seven regions could be directly compared: the MCX, CG, CB, MED, PONS, HP, and SN.

Where separation was achieved, PLS-DA VIP scores were used to identify regions that contributed most to separation; using this information, regions were removed from the PCA until the fewest number of regions required for separation was identified.

The potential effects of α-synuclein Braak staging on metal concentrations in PD cases and PDD controls were determined by performing linear regressions on the averaged concentrations of each metal across each sample across all regions and secondarily for individual regions.

## Results and discussion

### Cohort characteristics

Brain tissues were obtained from a total of 9 PD cases and 15 controls; the higher number of controls was due to insufficient quantities of tissue being available for some brain regions for some controls. PD cases received a clinical diagnosis of PD during their lifetime and a neuropathological diagnosis of PD based on α-synuclein Braak staging at postmortem; all controls were free from any neurodegenerative disease or cognitive impairment until their death and had an α-synuclein Braak stage of zero. In the overall cohort, cases and controls were matched for sex, PMD, tau Braak stage, and brain weight (see Table 1); however, PD cases were somewhat younger at the time of death than controls (76.6 vs. 85.6 years, *P* = .002). All cases and controls had a tau Braak stage of II or less. The same patterns were seen when looking at each of the brain regions individually (see Table S3).

**Table 1.** Overall cohort characteristics

Cohort	Age at death (years)	Sex	PMD (hours)	Tau Braak stage	α-syn Braak stage	Brain weight (g)
**All samples**						
Controls (*n* = 15)	85.6 ± 7.6 (71–99)	10 male (69%)	31.4 ± 21.6 (11–85)	II (0–II)	0	1218.1 ± 128.8 (989–1352)
PD cases (*n* = 9)	76.6 ± 4.7 (69–83)*	6 male (67%)	17.9 ± 7.1 (9–26)	II (0–II)	IV/V (III–VI)^****^	1240.3 ± 125.8 (1083–1380)^a^

This table shows mean ± SD (range) for age, sex, PMD, and brain weight and mode (range) for Braak staging. ^a^Data not available for every donor. *P < 0.05;^****^P < 0.0001.

### PD metallomics

The concentrations of eight essential metals (Na, Mg, K, Ca, Mn, Fe, Cu, and Zn) and the metalloid Se were measured by ICP-MS and compared using multiple Mann–Whitney *U* tests across seven brain regions, including the CG, HP, LC, MCX, MED, MCX, and SN.

**Figure 1.**
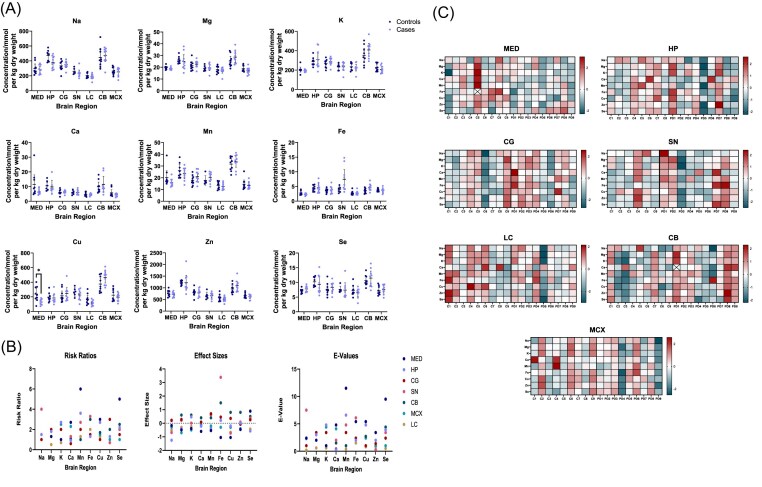
Metallomic analysis of PD cases and controls. (A) Case-control comparison of metal concentrations in PD cases and controls. Graphs show mean + 95% CIs; *P < .05. (B) RRs, effect sizes, and *E*-values for each region. (C) Heat maps showing *Z*-scores of individual cases and controls for each region.

No significant case-control differences were observed in Na, Mg, K, Ca, Mn, Fe, Zn, or Se levels in any region (see Table 2 and Fig. 1). The only significant metallic alteration observed was decreased Cu levels in the MED in PD cases compared to controls (237.6 vs. 129.1 μmol/kg; *q*-value = 0.03; *S*-value = 5.1; *E*-value = 5.4; RR = 3.0; effect size = −1.05). Concentrations of Cu were fairly consistent across regions in controls, with the only exception being significantly higher Cu in the CB compared to the LC (mean 374.7 vs. 164.7 μmol/kg, *P* = .0013; see Fig. S1). Cu levels were similarly high in the CB in cases, being significantly higher than in the MED, HP, SN, and LC (465.7, 129.1, 183.8, 129.1, and 115.9 μmol/kg, respectively; *P* = .0001, .04, .04, .0001, and <.0001, respectively; see Fig. S2). Regional comparisons for all metals for both cases and controls are presented in Figs S1 and S2.

**Table 2.** Metallomic analysis of PD cases and controls

**(a)**	**MED**	**Controls**	**Cases**	***P*-value**	***S*-value**	***E*-value**	**RR**	**RR lower CI *E*-value**	**RR upper CI *E*-value**	**Effect size**
	Na	298.1 ± 79.5 (237.0–359.3)	285.0 ± 44.9 (226.5–343.5)	1.0	0.0	2.4	1.5	0.32	6.9	−0.17
	Mg	19.6 ± 1.6 (18.4–20.8)	18.8 ± 0.8 (17.8–19.9)	0.6	0.7	2.0	1.3	0.4	4.3	−0.49
	K	205.2 ± 33.0 (179.9–230.6)	192.4 ± 8.6 (181.2–203.7)	0.3	1.7	1.0	1.0	0.7	13.6	−0.39
	Ca	9.0 ± 4.3 (5.4–12.5)	6.4 ± 1.5 (4.6–8.2)	0.6	0.7	4.8	2.7	0.05	2.922	−0.60
	Mn	20.2 ± 7.6 (14.4–26.0)	16.1 ± 2.6 (12.8–19.4)	0.4	1.3	11.5	6.0	0.4	118.7	−0.54
	Fe	2.8 ± 0.7 (2.2–3.4)	2.2 ± 0.3 (1.9–2.6)	0.3	1.7	2.0	1.3	0.8	9.6	−0.75
	Cu	237.6 ± 103.3 (158.3–317.0)	129.1 ± 30.8 (89.1–169.2)	0.03	5.1	5.4	3.0	0.81	11.1	−1.05
	Zn	773.0 ± 137.9 (667.0–879.0)	709.9 ± 85.2 (599.0–820.8)	0.8	0.3	3.4	2.0	0.5	8.3	−0.46
	Se	7.1 ± 0.8 (6.5.7.7)	7.8 ± 0.8 (6.8–8.8)	0.8	0.3	9.5	5.0	0.7	34.7	0.89
**(b)**	**HP**	**Controls**	**Cases**	***P*-value**	***S*-value**	***E*-value**	**RR**	**RR lower CI *E*-value**	**RR upper CI *E*-value**	**Effect size**
	Na	471.9 ± 73.2 (410.7–533.1)	380.2 ± 64.2 (296.7–463.7)	0.2	2.3	2.3	1.5	0.5	4.3	−1.25
	Mg	26.2 ± 3.1 (23.7–28.8)	25.0 ± 5.7 (17.6–32.3)	0.5	1.0	3.0	1.8	0.1	3.0	−0.42
	K	287.8 ± 46.7 (248.8–326.9)	309.7 ± 78.4 (207.7–411.6)	1.0	0.0	4.8	2.7	0.3	20.8	0.47
	Ca	10.6 ± 3.4 (7.4–13.8)	9.8 ± 4.4 (5.0–14.6)	1.0	0.0	0.3	0.9	0.1	11.0	−0.22
	Mn	26.1 ± 6.4 (19.4–32.8)	23.4 ± 4.3 (17.8–28.9)	0.70	0.5	6.6	3.6	0.2	81.8	−0.43
	Fe	4.4 ± 1.0 (3.6–5.2)	4.6 ± 1.3 (2.9–6.2)	1.0	0.0	2.0	1.3	0.3	6.0	0.21
	Cu	196.6 ± 65.6 (141.8–251.5)	183.8 ± 55.6 (111.4–256.1)	0.5	1.0	4.8	2.7	0.3	20.8	−0.20
	Zn	1220.3 ± 96.9 (1139.4–1301.3)	1222.0 ± 318.2 (808.1–1635.9)	0.7	0.5	2.0	1.3	0.3	6.1	0.02
	Se	10.2 ± 1.6 (8.9–11.6)	9.3 ± 2.5 (6.0–12.6)	0.5	1.0	3.9	2.2	0.6	8.4	−0.57
**(c)**	**CG**	**Controls**	**Cases**	***P*-value**	***S*-value**	***E*-value**	**RR**	**RR lower CI** ***E*-value**	**RR upper CI E-value**	**Effect size**
	Na	333.3 ± 70.2 (279.3–387.3)	349.0 ± 39.8 (297.3–400.7)	1.0	0.0	1.0	1.0	0.18	5.6	0.22
	Mg	21.5 ± 4.1 (18.4–24.7)	22.7 ± 2.5 (19.4–26.0)	0.6	0.7	3.4	2.0	0.2	18.3	0.28
	K	266.1 ± 43.3 (232.8–299.4)	286.3 ± 31.8 (244.9–327.7)	0.5	1.0	3.4	2.0	0.5	8.3	0.47
	Ca	6.1 ± 1.7 (4.8–7.5)	6.3 ± 0.9 (5.2–7.4)	0.9	0.2	0.4	0.6	0.62	5.1	0.09
	Mn	17.9 ± 4.2 (14.6–21.2)	20.9 ± 4.0 (15.7–26.1)	0.7	0.5	3.4	2.0	0.5	8.3	0.70
	Fe	3.5 ± 0.7 (3.0–4.1)	3.8 ± 1.0 (2.6–5.1)	1.0	0.0	2.4	1.5	0.3	6.9	0.45
	Cu	214.5 ± 84.7 (149.4–279.6)	245.1 ± 80.8 (139.9–350.3)	0.5	1.0	1.0	1.0	0.18	5.6	0.36
	Zn	802.7 ± 166.4 (674.8–930.6)	789.0 ± 128.4 (621.9–956.1)	1.0	0.0	1.4	3.0	0.3	23.7	−0.08
	Se	7.7 ± 2.1 (6.1–9.3)	8.3 ± 1.5 (6.3–10.2)	0.8	0.3	2.4	1.5	0.3	6.9	0.26
**(d)**	**SN**	**Controls**	**Cases**	***P*-value**	***S*-value**	***E*-value**	**RR**	**RR lower CI** ***E*-value**	**RR upper CI *E*-value**	**Effect size**
	Na	260.7 ± 38.6 (220.2–301.2)	234.4 ± 43.1 (178.4–290.5)	0.6	0.7	7.5	4.0	0.30	80.7	−0.68
	Mg	19.2 ± 2.3 (16.8–21.6)	19.7 ± 2.6 (16.3–23.1)	0.7	0.5	3.4	2.0	0.3	15.0	0.21
	K	232.6 ± 32.5 (198.5–266.7)	233.1 ± 38.6 (182.9–283.2)	0.8	0.3	3.4	2.0	0.3	15.0	0.01
	Ca	5.9 ± 1.4 (4.4–7.3)	6.0 ± 1.3 (4.3–7.7)	1.0	0.0	2.0	1.3	0.2	11.6	0.07
	Mn	17.7 ± 3.8 (13.7–21.7)	20.1 ± 3.5 (15.5–24.6)	0.7	0.5	4.8	2.7	0.4	18.4	0.61
	Fe	3.9 ± 1.0 (2.8–5.0)	7.4 ± 3.3 (3.2–11.7)	0.2	2.3	6.1	3.3	0.5	21.9	3.38
	Cu	266.9 ± 57.9 (206.2–327.7)	224.9 ± 56.7 (151.1–298.7)	0.60	0.7	4.8	2.7	0.4	18.4	−0.73
	Zn	658.8 ± 150.5 (500.8–816.8)	671.7 ± 102.8 (538.0–805.4)	0.8	0.3	0.3	0.7	0.1	8.7	0.09
	Se	7.6 ± 1.1 (6.5–8.7)	8.0 ± 1.2 (6.5–9.5)	0.9	0.2	3.4	2.0	0.3	15.0	0.38
**(e)**	**LC**	**Controls**	**Cases**	***P*-value**	***S*-value**	***E*-value**	**RR**	**RR lower CI** ***E*-value**	**RR upper CI E-value**	**Effect size**
	Na	204.2 ± 32.2 (179.4–229.0)	187.7 ± 28.3 (150.9–224.4)	1.0	0.0	2.4	1.5	0.3	6.9	−0.51
	Mg	18.1 ± 3.0 (15.8–20.4)	17.2 ± 2.2 (14.3–20.1)	0.9	0.2	0.4	0.5	0.1	4.6	−0.30
	K	234.4 ± 39.5 (204.1–264.8)	224.5 ± 34.5 (179.6 ± 269.4)	1.0	0.0	0.3	0.7	0.1	3.1	−0.25
	Ca	4.7 ± 1.3 (3.6–5.7)	4.5 ± 0.6 (3.8–5.3)	0.9	0.2	1.0	1.0	0.07	13.6	−0.12
	Mn	13.5 ± 3.1 (11.1–15.9)	12.6 ± 2.2 (9.8–15.5)	0.7	0.5	2.0	1.3	0.5	29.2	−0.29
	Fe	2.8 ± 0.7 (2.2–3.3)	2.9 ± 0.5 (2.2–3.6)	1.0	0.0	3.4	2.0	0.2	18.3	0.21
	Cu	164.7 ± 72.6 (108.9–220.5)	115.9 ± 28.7 (78.5–153.3)	0.2	2.3	2.0	1.3	0.2	2.4	−0.67
	Zn	571.3 ± 144.9 (459.9–682.6)	508.2 ± 87.6 (394.3–622.1)	0.8	0.3	2.0	1.3	0.4	4.3	−0.44
	Se	7.3 ± 1.8 (5.9–8.7)	6.5 ± 1.3 (4.8–8.2)	0.9	0.2	3.4	2.0	0.2	18.3	−0.43
**(f)**	**CB**	**Controls**	**Cases**	***P*-value**	***S*-value**	***E*-value**	**RR**	**RR lower CI** ***E*-value**	**RR upper CI *E*-value**	**Effect size**
	Na	444.1 ± 134.6 (340.6–547.6)	475.1 ± 82.2 (368.1–582.0)	1.0	0.0	2.4	1.5	0.3	6.9	0.2
	Mg	25.3 ± 5.2 (21.3–29.3)	28.7 ± 4.4 (22.9–34.4)	0.6	0.7	3.4	2.0	0.5	8.3	0.6
	K	352.0 ± 96.0 (278.2–425.8)	407.5 ± 66.2 (321.5–493.6)	0.5	1.0	3.4	2.0	0.5	8.3	0.6
	Ca	9.9 ± 4.2 (6.6–13.1)	11.5 ± 5.5 (4.9–18.1)	0.9	0.2	1.5	1.1	0.2	6.2	0.4
	Mn	31.6 ± 5.8 (27.1–36.1)	33.8 ± 4.8 (27.6–40.1)	0.7	0.5	1.0	1.0	0.3	3.7	0.4
	Fe	3.5 ± 0.8 (3.0–4.1)	4.7 ± 1.0 (3.5–6.0)	0.2	2.3	5.4	3.0	0.8	11.1	1.5
	Cu	374.7 ± 108.3 (291.5–457.9)	465.7 ± 76.8 (365.8–565.6)	0.1	3.3	2.7	1.7	0.6	5.0	0.8
	Zn	946.6 ± 187.7 (802.3–1090.9)	1092.1 ± 194.3 (839.3–1345.0)	0.8	0.3	3.4	2.0	0.5	8.3	0.8
	Se	10.6 ± 1.8 (9.2–12.0)	11.5 ± 2.4 (8.4–14.6)	0.9	0.2	4.4	2.5	0.6	9.7	0.5
**(g)**	**MCX**	**Controls**	**Cases**	***P*-value**	***S*-value**	***E*-value**	**RR**	**RR lower CI** ***E*-value**	**RR upper CI *E*-value**	**Effect size**
	Na	265.0 ± 49.2 (227.2–302.9)	252.2 ± 45.0 (193.6–310.7)	1.0	0.0	2.4	1.5	0.3	6.9	−0.3
	Mg	18.8 ± 2.5 (16.9–20.8)	17.1 ± 2.3 (14.2–20.1)	0.6	0.7	3.4	2.0	0.5	8.3	−0.7
	K	228.0 ± 39.4 (197.8–258.3)	206.9 ± 29.2 (168.9–244.9)	0.5	1.0	4.4	2.5	0.6	9.7	−0.5
	Ca	4.4 ± 0.5 (3.9–4.9)	4.3 ± 0.8 (3.3–5.3)	0.9	0.2	4.1	2.3	0.3	17.9	−0.1
	Mn	14.6 ± 4.7 (11.0–18.1)	13.5 ± 2.4 (10.4–16.6)	0.9	0.2	3.4	2.0	0.2	18.3	−0.2
	Fe	3.8 ± 0.5 (3.4–4.2)	3.7 ± 0.7 (2.8–4.5)	0.9	0.2	2.4	1.5	0.3	6.9	−0.3
	Cu	223.3 ± 81.3 (160.8–285.7)	195.2 ± 41.9 (140.6–249.8)	0.5	1.0	2.4	1.5	0.3	6.9	−0.3
	Zn	666.2 ± 106.5 (584.3–748.0)	619.3 ± 79.9 (515.4–723.2)	0.9	0.2	1.0	1.0	0.2	5.6	−0.4
	Se	7.2 ± 1.5 (6.0–8.3)	7.6 ± 1.2 (6.0–9.2)	0.9	0.2	1.0	1.0	0.3	3.7	0.3

Tables (a)–(g) show results of metallomic analysis of PD cases and controls. Units for Na, Mg, K, Ca, and Fe are mmol/kg dry weight and unis for Mn, Cu, Zn, and Se are µmol/kg dry weight. Case-control differences were determined by multiple Mann–Whitney *U* test with 10% FDR correction; *P*-value < .05 was considered significant. CI, confidence interval.

Multiple linear regressions were performed to investigate any potential confounding effects of age, tau Braak stage, and PMD on metal concentrations, firstly on overall metal concentrations averaged over all brain regions for each sample, and then on metal concentrations in individual regions. α-synuclein Braak stage was excluded from the analysis at this stage due to all controls being stage 0, leaving insufficient numbers at higher stages for analysis.

**Figure 2.**
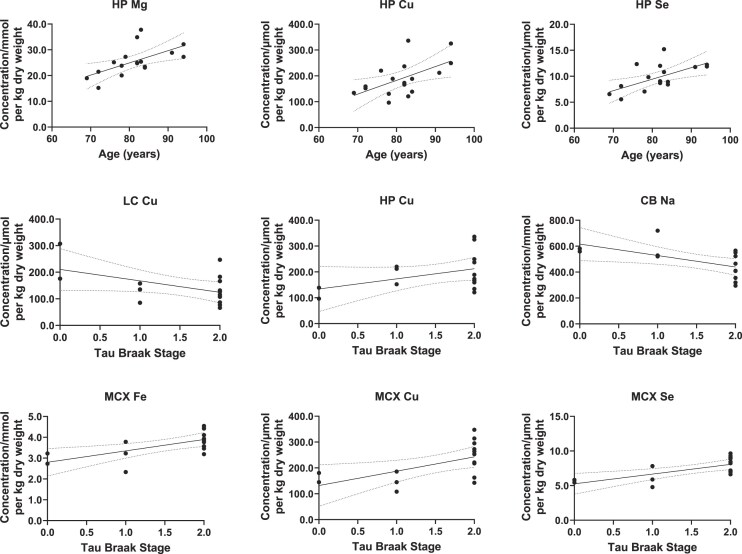
Linear regression of regional metal concentrations with age and tau Braak stage. This figure shows individual significant correlations (*P* < .05) between regional metal concentrations and tau Braak stage or age, as determined by multiple linear regression. No significant correlations were observed between PMD and regional metal concentrations.

Concerning averaged metal levels (averaged across all regions for each sample), there were no significant effects of age, tau Braak stage, or PMD on any metal (see Fig. S3). With regards to individual regions, age had a positive correlation with HP Mg [F (1, 11) = 7.5; *P* = .02; DF = 1], Cu [F (1, 11) = 9.0; *P* = .012; DF = 1], and Se [F (1, 11) = 8.7; *P* = .013; DF = 1] (see Fig. 2), with no significant relationships in any other region; tau Braak stage had a positive correlation with HP Cu [F (1, 11) = 7.3; *P* = .02; DF = 1] and MCX Fe [F (1, 11) = 6.7; *P* = .03; DF = 1], Cu [F (1, 11) = 5.8; *P* = .04; DF = 1], and Se [F (1, 11) = 10.2; *P* = .009; DF = 1] and a negative correlation with HP Se [F (1, 11) = 8.7; *P* = .013; DF = 1] and LC Cu [F (1, 11) = 5.1; *P* = .045; DF = 1]. No significant correlations with PMD were found for any metal in any brain region.

### PD vs. PDD

**Figure 3.**
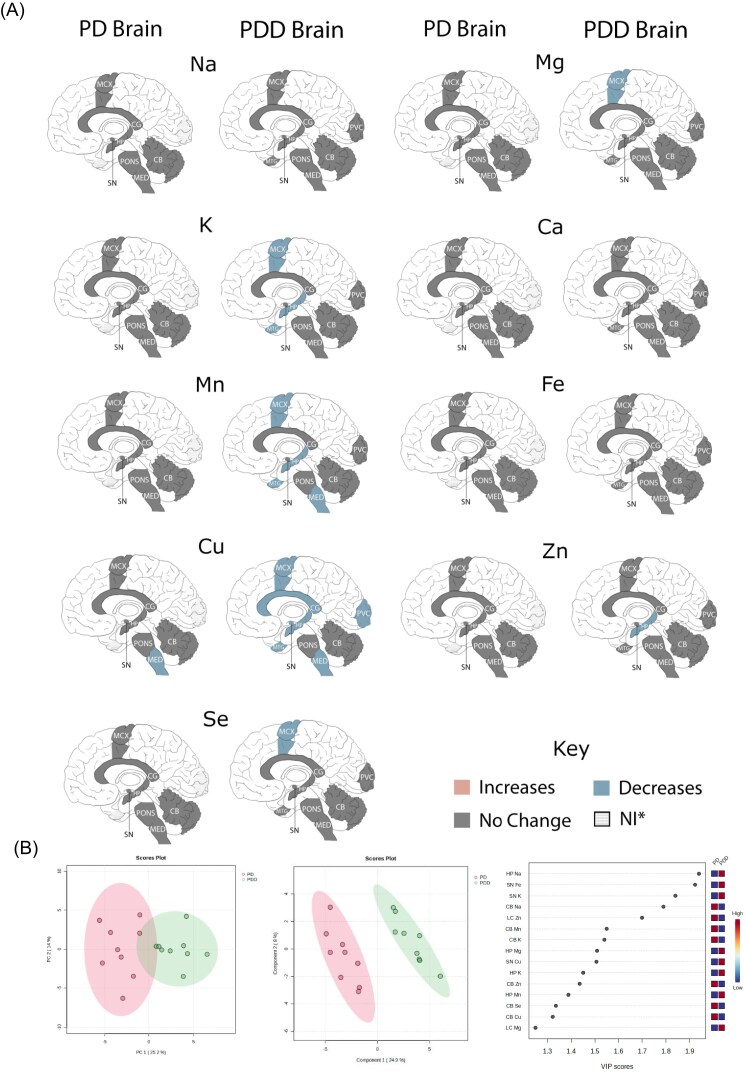
PD and PDD metals comparison. (A) Direct comparison of metallomic findings in PD and PDD cases. Areas shaded in grey denote no observed case-control changes; areas shaded in blue denote lower metal concentrations in cases than in controls; no regions showed increased metal concentrations in cases compared to controls in either PD or PDD. Areas shaded with dots were only investigated in one of either PD or PDD, and so could not be directly compared. (B) Separation of PD and PDD cases by PCA (left) and PLS-DA (middle); PD cases are shown in pink, and PDD cases are shown in green. VIP scores for PLS-DA are shown on the right; regional metals with a VIP score >1 were considered to contribute significantly to PD–PDD separation on PLS-DA.

The lack of perturbed metal levels in PD cases is striking in comparison to the numerous changes previously observed in the PDD brain [22], where Cu was decreased in seven of nine investigated regions (SN, CG, HP, MED, PVC, middle temporal gyrus (MTG), CB, MCX); decreased Mn was present in the SN, HP, MED, MTG, and MCX; K was decreased in the HP, MTG, and MCX; Mg was decreased in the MCX; Zn was decreased in the HP; and Se was decreased in the MCX. The only shared observation between the two conditions is decreased Cu in the MED, one of the earliest regions affected by Lewy body pathology in PD/PDD. Both PCA and PLS-DA separated PD and PDD cases based on these metallic data, with PLS-DA separation being contributed to mostly strongly by HP Na, SN Fe, and SN K data (see Fig. 3B).

PDD cases had a higher α-synuclein Braak stage in comparison to PD cases (see Table 3 and see Table S2 for individual PDD case/control cohort data); due to this, the relationship between metal levels and α-synuclein Braak stage was assessed using linear regression of cases from both the PD and PDD cohorts. PD and PDD cases did not show significant differences in age, sex distribution, or PMD (see Table 3).

**Table 3.** PDD cohort characteristics

Cohort	Age at death (years)	Sex	PMD (hours)	Tau Braak stage	α-syn Braak stage	Brain weight (g)
**All Samples**						
Controls (*n* = 9)	87.6 ± 5.2 (84–91)	4 male (44%)	26.7 ± 11.2 (20–34)	II (I–III)	0	1133.3 ± 158.1 (1030–1236)
PDD cases (*n* = 9)	77 ± 8.3 (72–82)**	5 male (56%)	28.6 ± 14.3 (19–38)	II (0–IV)	VI (V–VI)^****^	1299.9 ± 197.3 (1171–1429)

This table shows mean ± SD (range) for age, sex, PMD, and brain weight and mode (range) for Braak staging.**P < .01; ^****^P < .0001.

**Figure 4.**
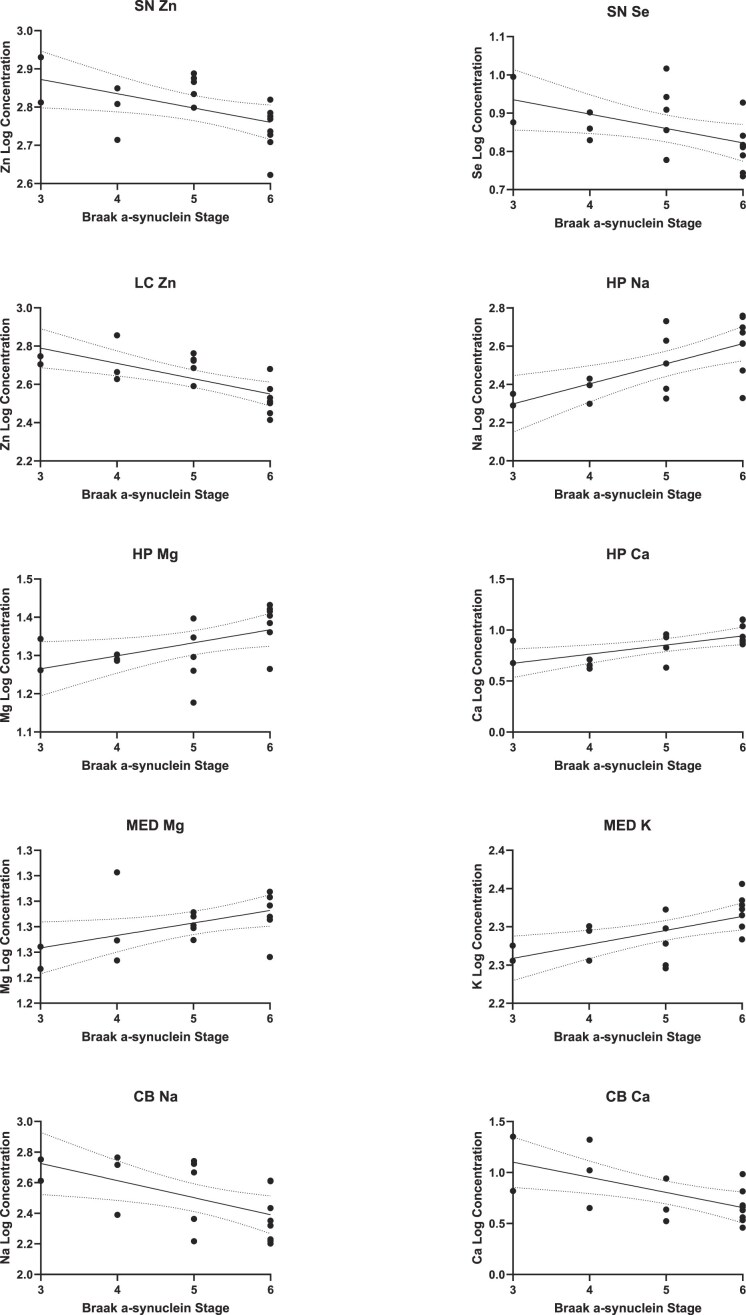
Correlations of regional metal concentrations with α-synuclein Braak stage. This figure shows significant correlations between α-synuclein Braak stage and regional metals as determined by multiple linear regression (*P* < .05).

Overall metal concentrations did not show any significant correlation with α-synuclein Braak stage (see Fig. 4); however, several significant correlations were observed when analyzing metal–Braak relationships in individual regions, including positive correlations with HP Na [F (1, 16) = 11.9; *P* = .003], HP Mg [F (1, 16) = 5.5; *P* = .03], HP Ca [F (1, 16) = 10.0; *P* = .007], MED Mg [F (1, 16) = 5.4; *P* = .03], and MED K [F (1, 16) = 9.4; *P* = .008] and negative correlations with SN Zn [F (1, 16) = 6.0; *P* = .03], SN Se [F (1, 16) = 5.3; *P* = .03], LC Zn [F (1, 16) = 14.6; *P* = .002], CB Na [F (1, 16) = 7.2; *P* = .02], and CB Ca [F (1, 16) = 8.7; *P* = .0010; see Fig. 4]. No significant correlations were found between α-synuclein Braak stage and Cu, Mn, or Fe in any region. Furthermore, no significant correlation was found between tau Braak stage and any metal in any region, showing a significant case-control difference in either PD or PDD (i.e. MED Cu in PD or, e.g. MCX Mg, HP Zn, or MCX Se in PDD).

**Figure 5.**
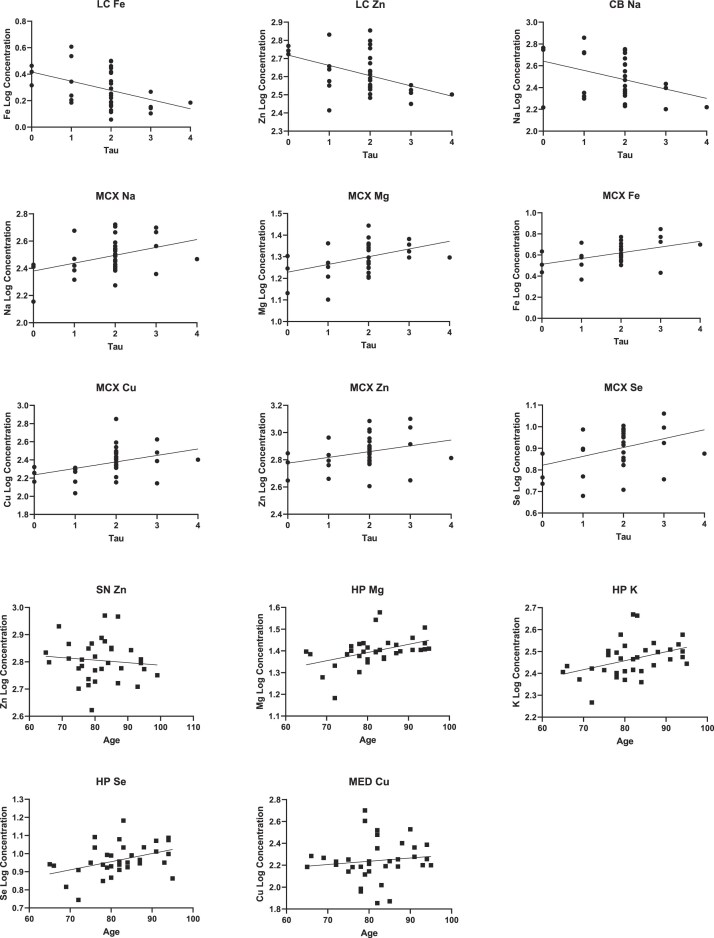
Correlations of regional metal concentrations with tau Braak stage and age. This figure shows significant correlations between tau Braak stage or age and regional metals in combined cohort, as determined by multiple linear regression (*P* < .05).

In addition to assessing the relationships between metal levels and α-synuclein Braak staging, an analysis of the effects of age and tau Braak stage was also carried out using the larger combined cohort of both PD and PDD cases and controls using multiple linear regression. All associations between age/tau and metal levels observed in the PD cohort were also found in the combined cohort; in addition, negative correlations between LC Fe [F (1, 29) = 4.6; *P* = .04] and Zn [F (1, 29) = 5.6; *P* = .02] and age and SN Zn [F (1, 26) = 4.329; *P* = .048], as well as positive correlations between age and HP K [F (1, 29) = 10.0; *P* = .004] and MED Cu [F (1, 29) = 5.3; *P* = .03], were also observed (see Fig. 5). An analysis of the effects of sex on metal concentrations, taking into account case/control status, using two-way ANOVA found a statistically significant effect of sex on CG Mn [F (1, 30) = 8.2; *P* = .008], CG Cu [F (1, 30) = 6.0; *P* = .02], and HP Mn [F (1, 29) = 4.9; *P* = .04], although correction by Tukey’s multiple comparisons test found no statistically significant difference between any individual groups (see Figs S4 and S5).

This study has revealed a striking lack of alterations in metallic levels in the PD brain in comparison to the PDD brain at postmortem; this may suggest that individuals with resistance to metallomic dysfunction in the brain may be more resilient to cognitive decline. In particular, despite decreased Cu in the MED being the only significant finding in any of the brain regions studied here, the widespread Cu decreases found throughout the PDD brain were almost completely absent in the PD brain, with none of the other metallomic alterations found previously in the PDD brain being observed here in any investigated region of the PD brain. A similar pattern can also be seen when comparing the PD brain to other neurodegenerative diseases where cognitive decline is present, such as AD [25] and dementia with Lewy bodies (DLB; [26]), in which we have also previously observed widespread Cu decreases, alongside other metallic alterations including localized decreases in Mg, K, Mn, Zn, and Se and increases in Na and Fe levels, and Huntington’s disease (HD), in which we have observed widespread decreases in Se levels [24].

It is perhaps one of the most striking observations of the current study that the widespread decreases in Cu levels previously observed in not only the PDD but also the AD and DLB brain were almost completely absent in the PD brain, with the exception of the MED. This suggests that although some Cu alterations may also be present in the absence of cognitive decline, this is much more widespread throughout the brains of individuals with PD who go on to develop concurrent dementia; a similar pattern is also seen in other neurodegenerative diseases characterized by the presence of cognitive decline, including AD [25] and DLB [26]. A study of Cu in the inferior temporal cortex, mid-frontal cortex, anterior cingulate, and CB of the AD brain found that higher composite brain Cu levels were associated with slower cognitive decline and lower levels of global AD neuropathology, with higher dietary levels of Cu also being associated with slower cognitive decline, despite not being directly associated with brain Cu levels themselves [27]. Another study found similar associations between serum Cu levels and MMSE scores in individuals with AD, even when levels remained within what is considered a healthy range [28]. Similar studies examining the cognitive profile of individuals with PD/PDD could establish if a similar pattern is present in these conditions. Reduced Mn levels have also been observed in individuals with both MCI and AD [29], with increased dietary intake of Mn associated with better cognitive scores [30]. Zn deficiency has been found to exacerbate cognitive decline in mouse models of HD [31] and AD [32] and has been reported to be associated with the development and speed of conversion to dementia in PD patients [33]. Taken together, there is an apparent overall lack of dysregulation of essential metal levels observed in the PD brain that has been seen to be present in the PDD, AD, and DLB brain.

It is interesting to note that the MED, the only region to show any metallic alterations in PD, is one of the earliest regions of the brain affected in the usual neuropathological progression of PD, with PD Braak stage I being characterized exclusively by α-synuclein inclusions within the dorsal IX/X motor nucleus and/or intermediate reticular zone of the MED [23]. It may be that as the earliest affected brain region in most PD cases, the MED is particularly vulnerable to Cu dysregulation. Cu has been found to interact with α-synuclein, with some studies reporting that Cu binding increases the aggregation and misfolding of α-synuclein and promotes the formation of fibrils [34–36]. Such interactions are not reflected by the linear regression analyses conducted in the current study, in which the α-synuclein Braak stage was not found to correlate with Cu levels in any brain region; however, this analysis may have been limited by the relatively low number of samples representing some stages, and so further investigation of this potential correlation should be conducted in studies with larger sample sizes. However, it is possible that the differences in Cu alterations and cognitive decline between PD and PDD cases are entirely independent of α-synuclein pathology.

Another potential contributing factor to the difference in Cu observations between PD and PDD, AD, and DLB may be the presence of differing levels of tau pathology. Cu has been reported to interact with tau protein and has been suggested to promote its hyperphosphorylation and fibrilization [37, 38]. Although some level of tau pathology is commonly seen in all of these conditions, PDD, AD, and DLB are more likely to be classified with the higher tau Braak stages III–VI than PD [39]; furthermore, total tau levels in CSF have also been shown to correlate with measures of cognitive decline in PD [40]. These patterns are in line with the results of the regression analyses performed in the current study, where Cu levels were found to correlate with the tau Braak stage in multiple regions in the PD brain, including the LC, HP, and MCX. However, these correlations were not replicated in the combined PD–PDD case cohort. Although the PDD cohort examined here had the same average Braak stage, it had a wider range, including higher stages than those seen in PD cases; however, as in the case of α-synuclein, it is possible that the analysis of potential tau Braak–Cu correlations was limited here by the small sample size and subsequent low numbers of samples at some Braak stages. As such, further analyses with a larger number of both PD and PDD cases stratified across a wider range of tau Braak stages are required to elucidate any potential correlation between tau neuropathology and cerebral Cu levels. Furthermore, although age was found to correlate with HP Cu in the PD cases, this finding was not replicated in the combined PD–PDD case group, and there was no significant difference in age at death between PD and PDD cases that could explain the differences in Cu observations present.

Copper is an essential trace metal that performs a variety of important functions in the brain and throughout the rest of the body. As such, there are many mechanisms by which Cu dysregulation might contribute to pathogenesis in PD/PDD and other neurodegenerative diseases. Perhaps the most important role of Cu in the brain is as a cofactor of the antioxidant superoxide dismutase 1 (SOD1), which is responsible for clearance of free superoxide radicals via their conversion to oxygen and hydrogen peroxide. Insufficient levels of Cu may therefore result in a lowering in the antioxidative capacity of the brain and consequentially increased oxidative stress, as is observed in PD [41]. SOD1 aggregates, similar to those observed in SOD1-associated familial amyotrophic lateral sclerosis (fALS), have also been observed in the PD brain, exhibiting both misfolding and decreased Cu-binding, independent of any mutations in the SOD1 gene [42]. This combination of protein misfolding and lowering of a metallic cofactor could well lead to SOD1 being unable to perform its antioxidant roles effectively, resulting in a reduced antioxidant response.

Also striking is the lack of Fe changes found in the PD brain; although this is in accordance with the lack of Fe alterations observed in our previous analysis of the PDD brain, many investigations carried out by other research groups have reported Fe changes in several regions of the brain, including the SN [6–14], GP [8], and PUT [12]. However, other groups have reported no changes in the SN [15, 16, 43, 44] and HP [16], as well as in other brain regions [6, 9, 12, 14–16]. These discrepancies may simply reflect heterogeneity in Fe levels across individuals with PD, or may be the result of other factors such as differences in methodologies; e.g. iron is often measured in the brain *in vivo* using MRI. Such methods have the obvious advantage of allowing us to measure Fe levels within a living person’s brain; however, they are much less sensitive than methods such as ICP-MS, and cannot be used to accurately quantify absolute Fe levels. *In vivo* and postmortem Fe levels may also reflect differences in concentrations at different stages of the disease, with postmortem tissues representing the very end stage and *in vivo* measurements being viable throughout many different stages of disease (although cognitive symptoms may make it difficult for a patient to undergo MRI at later stages).

Another potential explanation for some of the differing reports on Fe in PD is the type of Fe being measured; e.g. a study by Wypijewska et al. found that, while total Fe levels were not increased in the SN of their cohort, they did observe increased levels of labile Fe [45]. Koziorowski et al. found increased levels of heavy-chain ferritin, but decreased levels of light-chain ferritin, in the PD SN [46], with Bartzokis et al. observing increased ferritin-bound Fe in early-onset PD and a reduced ferritin-binding capacity in later-onset PD in the SN [47]. In the case of the SN, results may also vary depending on whether the SN is investigated as a whole (as performed here) or whether it is further subdivided; e.g. some studies have reported selective Fe increases within the SN pars compacta, without statistically significant increases being observed in the SN pars reticulum [48–50]. Further studies with larger cohorts investigating the levels of different types of Fe across different subregions of the SN would be required to determine how these factors may influence Fe findings in the PD brain.

It should also be noted that the current study investigated whole-tissue metal levels without studying the cellular or subcellular localization of metals. Fe increases and Cu decreases have been previously observed within the soluble cellular fraction, without concurrent changes in the membrane-associated or insoluble fractions [9], and both Lewy bodies [51] and amyloid-β inclusions [52, 53] have been reported to sequester metals such as Fe and Cu. Furthermore, in one report on the PD brain, Fe was found to be unchanged in SN astroglial or ferritin-positive oligodendroglial cells while being increased in other cell types, and ferritin was found to be depleted in PD oligodendroglial cells [54]. As such, whole-tissue investigations such as the one carried out in the current study may not reflect more localized metallic alterations in the PD brain.

## Conclusions

PD without dementia shows a striking lack of metallic involvement in comparison to PDD, manifesting few of the many metallomic alterations present in the latter. Many of these alterations have also been observed in other neurodegenerative diseases characterized by the presence of dementia, including AD and DLB. Further studies should now be performed to determine why some individuals appear to show this resistance and investigate how this information might be used to induce higher resistance to cognitive decline in individuals susceptible to the development of dementia.

## Supplementary Material

mfaf006_Supplemental_Files

## Data Availability

All raw data for individual runs, as well as mean values of triplicate runs, are included in Supplementary Material B; this also includes blank values, standard curve values, tissue wet and dry weights, and indication of if values were excluded from analysis as outliers or due to high blank values. Concentrations are given in both mmol per kg/nmol per kg and ug/l. Values are given for mean averages, standard deviations, confidence intervals, and Mann–Whitney *U* test results. Details of all software used for analyses is included in the methods section.
