# Supplementary material for: Perturbations in levels of essential metals less severe in Parkinson’s disease without dementia than in Parkinson’s disease dementia
Source: Metallomics. 2025 Feb 11;17(3):mfaf006. doi: 10.1093/mtomcs/mfaf006 (PMC11895508; doi:10.1093/mtomcs/mfaf006)
Supplement: mfaf006_Supplemental_Files [file mfaf006_supplemental_files.zip › Suppl_Data A1.docx]

## Supplementary Table A1. Individual Characteristics of PD Cohort Donors

| **ID** | **Status** | **Age at Death (years)** | **Sex** | **Brain Weight (g)** | **PMD (hours)** | **Tau Braak Stage** | **α-Synuclein Braak Stage** | **SVD** | **Amyloid Angiopathy** | **Cause of Death** | **Comorbidities** | **Medications** |
| --- | --- | --- | --- | --- | --- | --- | --- | --- | --- | --- | --- | --- |
| **C01** | Control | 75 | M | 1256 | 17 | *N/A* | 0 | Mild-moderate | None | Aspiration Pneumonia, CVA | CVA, chest infection | *N/A* |
| **C02** | Control | 71 | M | 1334 | 29 | *N/A* | 0 | Mild | Mild | Liver cancer | IBS, asthma, liver cirrhosis, hepatocellular carcinoma and prostatism | *N/A* |
| **C03** | Control | 84 | F | 1124 | 22 | *N/A* | 0 | Mild | None | Old age | Hypothyroidism, polymyalgia rheumatica, osteoporosis, acute myocardial infarction, chronic kidney impairment | *N/A* |
| **C04** | Control | 84 | F | 1116 | 23 | 0 | 0 | Mild | None | Old age | *N/A* | *N/A* |
| **C05** | Control | 94 | F | 1352 | 24 | 2 | 0 | None | None | Heart failure | COD pneumonia, valvular heart disease, atrial fibrillation, left ventricular failure | *N/A* |
| **C06** | Control | 94 | M | 1351 | 11 | 2 | 0 | None | None | Old age, ischaemic heart disease | *N/A* | *N/A* |
| **C07** | Control | 83 | M | 1346 | 21 | 2 | 0 | None | None | Pulmonary fibrosis | Possible PART | *N/A* |
| **C08** | Control | 91 | M | 1338 | 18 | 2 | 0 | Mild | None | Unknown | Cholelithiasis, arthralgia of multiple joints, benign prostatic hypertrophy, acute cholecystitis, kyphoscoliosis | Simvastatin, dipyridamole, finasteride, aspirin |
| **C09** | Control | 83 | M | 1256 | 26 | 2 | 0 | None | None | Metastatic prostate cancer | Hypertension, cataracts, cellulitis, Raynaud's phenomenon | Morphine, midazolam, hyoscine butylbromide, haloperidol |
| **C10** | Control | 85 | M | 989 | 85 | 2 | 0 | None | None | Lobar pneumonia | Glaucoma, lumbar disc laminectomy, benign prostatic hypertrophy, aortic stenosis, solar keratosis, hearing loss, cataracts | Warfarin, finasteride, rivaroxaban, eye drops |
| **C11** | Control | 79 | M | 1331 | 79 | 2 | 0 | None | None | Brainstem stroke, bronchopneumonia | Hypertension, type II diabetes, ischaemic heart disease, thrombocytopenia, kidney cysts, malignant neoplasm on cheek, bilateral diabetic retinopathy | Tramadol, pregabalin, docusate |
| **C12** | Control | 82 | M | 1100 | 20 | 2 | 0 | Moderate | None | Hospital-acquired pneumonia, congestive heart failure, acute kidney injury, osteomyelitis | Type II diabetes, hypertension, osteoarthritis, congestive heart failure, chronic kidney disease, COPD, peripheral neuropathy, small cerebellar infarct | Spiriva, NovoMix, senna, spirolactone, furosemide, zopiclone, pregabalin, colecalciferol, bisoprolol, simvastatin |
| **C13** | Control | 91 | F | 1081 | 22 | 1 | 0 | None | None | Cardiac failure, hypotension, atrial fibrillation, aortic stenosis | Systemic vasculitis and Sjogren's syndrome, essential hypertension, severe aortic stenosis, congestive heart failure, angina, hyperthyroidism, ischaemic heart disease, hearing loss, chronic kidney disease | Mirtazapine, ranitide, hydroxychloroquine, ferrous fumarate, ascorbic acid, alendronic acid, loperamide |
| **C14** | Control | 90 | M | *N/A* | 34 | *N/A* | *N/A* | *N/A* | *N/A* | Chest infection, stroke | *N/A* | *N/A* |
| **C15** | Control | 99 | M | 1245 | 36 | 2 | 0 | Mild | None | *N/A* | PART | *N/A* |
| **C16** | Control | 82 | F | 1090 | 30 | 2 | 0 | *N/A* | *N/A* | *N/A* | PART | *N/A* |
| **PD1** | PD | 83 | F | 1288 | 24 | 2 | 5 | None | None | Myocardial infarction, coronary artery disease, metastatic lung cancer | Viral encepathalitis, anxiety, essential hypertension, angina, macular degeneration | cobeneldopa, bisoprolol, bendroflazide, nasonex, aspiritin, lansoprazole, bezafibrate, oxybutinin, adcal, digoxin, strontium |
| **PD2** | PD | 72 | M | 1380 | 26 | 1 | 5 | *N/A* | *N/A* | Stanford type A aortic dissection, Parkinson's disease | Chronic kidney disease, lentigo maligna, retinal haemorrhage, possible MSA | Sinemet, fludrocortisone, midodrine, movicol, levodopa, fibrogel |
| **PD3** | PD | 79 | F | 1083 | 9 | 2 | 6 | *N/A* | *N/A* | *N/A* | PART, CAA type II | *N/A* |
| **PD4** | PD | 76 | M | 1210 | 24 | 1 | 4 | *N/A* | *N/A* | Parkinson's disease | Pacemaker, prostatism, multiple fractures | Madopar, rasagiline, fludrocortisone, warfarin, levodopa |
| **PD5** | PD | 72 | M | *N/A* | 11 | 2 | 3 | *N/A* | *N/A* | *N/A* | *N/A* | *N/A* |
| **PD6** | PD | 78 | M | *N/A* | 11 | *N/A* | 4 | *N/A* | *N/A* | Aspiration pneumonia, Parkinson's disease | *N/A* | *N/A* |
| **PD7** | PD | 82 | F | *N/A* | 26 | 2 | 5 | Mild-moderate | None | Parkinson's disease | *N/A* | *N/A* |
| **PD8** | PD | 69 | M | *N/A* | 13 | 2 | 3 | Mild | Mild | Metastatic colon cancer, Parkinson's disease | *N/A* | *N/A* |
| **PD9** | PD | 78 | M | *N/A* | 17 | 0 | 4 | Mild | None | Parkinson's disease, old age | *N/A* | *N/A* |

Table shows all individual data for PD cases and controls, where made available by the brain bank. CAA = Cerebral amyloid angiopathy; COPD = Chronic obstructive pulmonary disease; CVA = Cerebrovascular accident; IBS = Irritable bowel syndrome; MSA = Multiple system atrophy; N/A = Information not available; PART = Primary age-related tauopathy ;PD = Parkinson’s disease; PMD = Post-mortem delay; SVD = Small vessel disease.

## Supplementary Table A2. Individual Characteristics of PDD Cohort Donors

| **ID** | **Status** | **Age at Death (years)** | **Sex** | **Brain Weight (g)** | **PMD (hours)** | **Tau Braak Stage** | **α-Synuclein Braak Stage** |
| --- | --- | --- | --- | --- | --- | --- | --- |
| **PDDC1** | Control | 82 | Female | 946 | 15 | 2 | 0 |
| **PDDC2** | Control | 95 | Female | 1126 | 28 | 3 | 0 |
| **PDDC3** | Control | 91 | Male | 1338 | 18 | 2 | 0 |
| **PDDC4** | Control | 85 | Male | 989 | 29 | 2 | 0 |
| **PDDC5** | Control | 79 | Male | 1331 | 25 | 2 | 0 |
| **PDDC6** | Control | 87 | Female | 978 | 15 | 2 | 0 |
| **PDDC7** | Control | 94 | Female | 1158 | 40 | 2 | 0 |
| **PDDC8** | Control | 88 | Female | 1033 | 23 | 3 | 0 |
| **PDDC9** | Control | 87 | Male | 1301 | 48 | 1 | 0 |
| **PDD1** | PDD | 65 | Male | 1642 | 39 | 0 | 5 |
| **PDD2** | PDD | 80 | Male | 1165 | 26 | 2 | 6 |
| **PDD3** | PDD | 75 | Male | 1399 | 48 | 1 | 6 |
| **PDD4** | PDD | 93 | Female | 1222 | 10 | 3 | 6 |
| **PDD5** | PDD | 66 | Male | 1371 | 27 | 4 | 6 |
| **PDD6** | PDD | 80 | Female | 1152 | 20 | 2 | 6 |
| **PDD7** | PDD | 80 | Male | 1402 | 31 | 2 | 5 |
| **PDD8** | PDD | 78 | Female | 1386 | 47 | 3 | 6 |
| **PDD9** | PDD | 76 | Female | 960 | 9 | 1 | 6 |

PDD = Parkinson’s disease dementia; PMD = Post-mortem delay

## Supplementary Table A3. Summaries of PD Cohort by Region

| **Cohort** | **Age at Death (years)** | **Sex** | **PMD (hours)** | **Tau Braak Stage** | **α-syn Braak Stage** | **Brain Weight (g)** |
| --- | --- | --- | --- | --- | --- | --- |
| **MED** | | | | | | |
| Controls (n = 9) | 84.4 ± 6.1  (75–94) | 6 Male  (66%) | 30.1 ± 19.1  (17–79) | II (0–II) | 0 | 1229.9 ± 111.9  (1090–1352) |
| PD Cases (n = 9) | 76.6 ± 4.7  (69–83)** | 6 Male  (66%) | 17.9 ± 7.1  (9–26) | II (0–II) | 4 (3–6)**** | 1240.3 ± 125.8  (1083–1380)^†^ |
| **HP** | | | | | | |
| Controls (n = 8) | 85.0 ± 7.2  (71–94) | 4 Male  (44%) | 22.4 ± 5.5  (11–30) | II (0–II) | 0 | 1210.4 ± 129.1  (1081–1352) |
| PD Cases (n = 9) | 76.6 ± 4.7  (69–83)* | 6 Male  (66%) | 17.9 ± 7.1  (9–26) | II (0–II) | 4 (3–6)**** | 1240.3 ± 125.8  (1083–1380)^†^ |
| **CG** | | | | | | |
| Controls (n = 9) | 86.3 ± 7.9  (71–94) | 6 Male  (66%) | 21.0 ± 5.2  (11–29) | II (0–II) | 0 | 1252.3 ± 127.4  (1081–1352) |
| PD Cases (n = 9) | 76.6 ± 4.7  (69–83)* | 6 Male (66%) | 17.9 ± 7.1  (9–26) | II (0–II) | 4 (3–6)**** | 1240.3 ± 125.8  (1083–1380)^†^ |
| **SN** | | | | | | |
| Controls (n = 6) | 86.7 ± 7.5  (69–83) | 6 Male  (66%) | 28.8 ± 22.1  (11–85) | II (0–II) | 0 | 1208.8 ± 131.8  (989–1352) |
| PD Cases (n = 9) | 76.6 ± 4.7  (69–83)** | 6 Male  (66%) | 17.9 ± 7.1  (9–26) | II (0–II) | 4 (3–6)**** | 1240.3 ± 125.8  (1083–1380)^†^ |
| **LC** | | | | | | |
| Controls (n = 9) | 86.4 ± 6.4  (75–94) | 5 Male  (56%) | 19.8 ± 4.0  (11–24) | II (0–II) | 0 | 1229.3 ± 121.8  (1081–1352) |
| PD Cases (n = 9) | 76.6 ± 4.7  (69–83)** | 6 Male  (66%) | 17.9 ± 7.1  (9–26) | II (0–II) | 4 (3–6)**** | 1240.3 ± 125.8  (1083–1380)^†^ |
| **CB** | | | | | | |
| Controls (n = 9) | 86.4 ± 6.4  (75–94) | 5 Male  (56%) | 19.8 ± 4.0  (11–24) | II (0–II) | 0 | 1229.3 ± 121.8  (1081–1352) |
| PD Cases (n = 9) | 76.6 ± 4.7  (69–83)** | 6 Male  (66%) | 17.9 ± 7.1  (9–26) | II (0–II) | 4 (3–6)**** | 1240.3 ± 125.8  (1083–1380)^†^ |
| **MCX** | | | | | | |
| Controls (n = 9) | 86.4 ± 6.4  (75–94) | 5 Male  (56%) | 19.8 ± 4.0  (11–24) | II (0–II) | 0 | 1229.3 ± 121.8  (1081–1352) |
| PD Cases (n = 9) | 76.6 ± 4.7  (69–83)** | 6 Male  (66%) | 17.9 ± 7.1  (9–26) | II (0–II) | 4 (3–6)**** | 1240.3 ± 125.8  (1083–1380)^†^ |

Table shows mean ± SD (range) for age, sex, PMD, duration of disease, and brain weight and mode (range) for Braak staging. ^†^Data not available for every donor.

## Supplementary Table A4. Comparison of PD and PDD Cohort Characteristics

| **Cohort** | **Age at Death (years)** | **Sex** | **PMD (hours)** | **Tau Braak Stage** | **α-syn Braak Stage** | **Duration of Disease (years)** | **Brain Weight (g)** |
| --- | --- | --- | --- | --- | --- | --- | --- |
| **Controls** | | | | | | | |
| PD | 85.6 ± 7.6  (71–99) | 10/15 Male (69%) | 31.4 ± 21.6  (11–85) | II  (0–2) | 0 | N/A | 1218.1± 128.8 (989–1352) |
| PDD | 87.6 ± 5.2  (79–95) | 4/9 Male  (44%) | 26.8 ± 11.2  (15–48) | II  (1–3) | 0 | N/A | 1133.3 ± 158.1 (946–1338) |
| **Cases** | | | | | | | |
| PD | 76.6 ± 4.7  (69–83) | 6/9 Male  (67%) | 17.9 ± 7.1  (9–26) | II  (0–II) | IV/V  (III–VI) | 7.0 ± 4.5  (3–13)^†^ | 1240.3 ± 125.8 (1083–1380)^†^ |
| PDD | 77.0 ± 8.3  (65–93) | 5/9 Male  (56%) | 28.6 ± 14.3  (9–48) | II  (0–IV) | VI  (V–VI) | 13.8 ± 6.1  (6–23) | 1299.9 ± 197.3 (960–1642) |

Table shows mean ± SD (range) for age, sex, PMD, duration of disease, and brain weight and mode (range) for Braak staging. ^†^Data not available for every donor.

Supplementary Figure A1: PD Control Regional Comparisons

*Figure shows regional metal concentrations, with inter-regional differences determined by one-way ANOVA (p < 0.05). * p < 0.05, ** p < 0.01, *** p < 0.001, **** p < 0.0001. MED = Medulla; HP = Hippocampus; CG = Cingulate gyrus; SN = Substantia nigra; LC = Locus coeruleus; CB = Cerebellum; MCX = Motor cortex.*

Supplementary Figure A2: PD Case Regional Comparisons

*Figure shows regional metal concentrations, with inter-regional differences determined by one-way ANOVA (p < 0.05). * p < 0.05, ** p < 0.01, *** p < 0.001, **** p < 0.0001. MED = Medulla; HP = Hippocampus; CG = Cingulate gyrus; SN = Substantia nigra; LC = Locus coeruleus; CB = Cerebellum; MCX = Motor cortex.*

Supplementary Figure A3: Linear Regression Graphs for Averaged Metal Concentrations vs Age

*Figure shows significant correlations between age and* *metal levels averaged across all regions, as determined by multiple linear regression (p < 0.05).*

Supplementary Figure A4: Linear Regression Graphs for Averaged Metal Concentrations vs Tau Braak Stage

*Figure shows significant correlations between tau Braak stage and metal levels averaged across all regions, as determined by multiple linear regression (p < 0.05).*

Supplementary Figure A5: Analysis of Sex Differences in Regional Metal Concentrations in PD, PDD, and Controls

*Figure shows results of two-way ANOVA analysing sex differences in metal concentrations in PD, PDD, and controls. Sex was initially found to have a significant overall effect on CG Mn, CG Cu, and HP Mn (p < 0.05), but correction with Tukey’s multiple comparison test showed no significant differences between any two individual groups (adjusted p-value > 0.05).*
